# Bilateral Retrobulbar Optic Neuritis as the First Manifestation of Neuro-Behçet Disease

**DOI:** 10.1155/2020/8834399

**Published:** 2020-10-06

**Authors:** Mohsen Jari, Taiiebeh Mohammadi, Ensiyeh Taheri

**Affiliations:** ^1^Department of Pediatric Rheumatology, Imam Hossein Children's Hospital, Isfahan University of Medical Sciences, Isfahan, Iran; ^2^Department of Pediatric, Imam Hossein Children's Hospital, Isfahan University of Medical Sciences, Isfahan, Iran

## Abstract

**Background:**

Behçet disease (BD) is a polygenic and chronic autoinflammatory multisystem vasculitis. Acute optic neuritis has been rarely reported in patients with BD, especially in children. *Case Presentation*. We reported an 8-year-old girl with a sudden visual loss and color vision impairment. The patient had a history of recurrent oral aphthous ulcers, genital ulcers, and chronic abdominal pain. On ophthalmic examination, anterior and posterior chambers and funduscopy of both eyes were normal. The results of laboratory tests for infectious and rheumatic diseases were normal. Brain magnetic resonance imaging and the result of cerebrospinal fluid analysis for oligoclonal bands and auto-antibodies were also normal. Pathergy skin test and human leukocyte antigens (HLA) B5 and HLA-B51 were positive. The patient was recognized as a case of BD-related bilateral retrobulbar optic neuritis and was treated by corticosteroids, azathioprine, colchicine, and infliximab.

**Conclusion:**

Retrobulbar optic neuritis may be the first manifestation of neuro-BD.

## 1. Introduction

Behçet disease (BD) is a chronic disease associated with venous and arterial vasculitis, represented by recurrent oral aphthous ulcers, genital ulcers, skin lesions, neurologic and ocular involvement, arthritis, and gastrointestinal (GI) involvement [1]. Ocular involvement, which is seen in 30%–60% of BD cases, is more commonly associated with panuveitis, which is usually bilateral. Iridocyclitis and posterior uveitis are ocular manifestations of BD. Retinal vasculitis, retinal detachment, and optic neuritis also occur rarely [2, 3]. Retrobulbar optic neuritis is one of the very rare manifestations of retinal involvement in BD cases, especially in children [4]. Here, we report BD-related bilateral retrobulbar optic neuritis, which is a very rare case of ocular involvement in children.

## 2. Case Presentation

An eight-year-old girl was admitted in the pediatric neurology ward of Imam Hossein Children's Hospital, Isfahan University of Medical Sciences, due to a sudden visual loss in both eyes and headache in the occipital area. The patient had no fever, and she did not have any symptoms of meningitis, including neck stiffness and Brudzinski and Kernig signs, in physical examinations. Cranial nerve examination was normal, visual acuity of the right eye was hand motion of 50 cm, and the left eye was 100 cm. The patient had visual impairment in recognizing green and red colors. On ophthalmic examination, anterior and posterior chambers and funduscopy of both eyes were completely normal. The patient had a history of recurrent oral aphthous ulcers (5-6 times a year), genital ulcers, chronic and vague abdominal pain, diarrhea, and intermittent constipation. She had been treated with intravenous immunoglobulin due to Kawasaki disease when she was three years old.

The results of laboratory tests including complete blood count, erythrocyte sedimentation rate, C-reactive protein, serum electrolytes, infectious diseases' serology for toxoplasmosis, cytomegalovirus, tuberculosis, and rheumatologic tests including antinuclear antibody, angiotensin-converting enzyme, antiphospholipid antibodies, and serum complement levels were normal. The result of Mantoux test was negative. Chest X-ray and brain magnetic resonance imaging (MRI) without contrast results were normal either. The result of cerebrospinal fluid analysis including cell, sugar, protein, smear, culture, oligoclonal bands, neuromyelitis optica (NMO) antibody, and myelin oligodendrocyte glycoprotein (MOG) was normal. Fecal calprotectin level was 252 *μ*g/mg (NL < 50). GI endoscopy and colonoscopy results were normal. The patient was transferred to the pediatric rheumatology ward. The result of the pathergy skin test, on the forearm area performed with intradermal needle 21 gauge, was positive. Both human leukocyte antigen (HLA) B5 and HLA-B51 tests were positive.

According to the criteria of the International Study Group for Behçet Disease, the patient was recognized as a case of BD-related bilateral retrobulbar optic neuritis, and the treatment started by prescribing pulse methylprednisolone 30 mg/kg/d (max 1 gr) for three days. Then, in the fourth day of treatment, intravenous infusion with infliximab 8 mg/kg was given. The patient was discharged on the fifth day of hospitalization with oral prednisolone 2 mg/kg/d, azathioprine 2 mg/kg/d, and colchicine 1 mg/d. After a one-week follow-up, the patient had no color vision impairment, and both eyes could see two meters away. Ocular exam through slit lamp and funduscopy was normal. She did not have headache and nausea. After a one-month follow-up, the patient had no color vision impairment, and ocular exam through slit lamp and funduscopy was also normal. Visual acuity of both was around 20/40. Infusion of infliximab 8 mg/kg was given, and oral prednisolone 1 mg/kg/d with azathioprine and colchicine with similar doses was continued. Three months after discharge, the patient did not have any complain about aphthous ulcers and GI system problems. Visual acuity of both eyes was 20/20, and ocular exam was normal. Azathioprine, colchicine, and infliximab infusion continued monthly. Oral prednisolone was tapered to 0.2 mg/kg/d.

## 3. Discussion

Behçet disease (BD) is a polygenic and chronic autoinflammatory multisystem vasculitis characterized by mucocutaneous, musculoskeletal, neurological, gastrointestinal, and ophthalmological lesions. Childhood-onset BD is uncommon, accounting for 3% to 7% of all cases. Prevalence in children is probably not more than 10% of the adult counterparts in Eastern Mediterranean countries [5].

BD rarely presents with acute optic neuritis. Few publications have reported an association between BD and optic neuropathy. Retrobulbar neuritis (optic neuritis) is a rare manifestation of neuro-Behçhet disease [4, 6]. The diagnosis in children is difficult as the disease is uncommon and clinically resembles other diseases, such as multiple sclerosis. Optic neuritis is a consideration whenever monocular or binocular blindness develops suddenly in a child. The initial feature in some children is pain in the eye, but for most, it is blurred vision, progressing within hours or days to partial or complete blindness. Visual acuity reduces to less than 20/200 in almost all affected children within 1 week. Visual-evoked response testing further confirms the diagnosis. Orbit MRI may reveal swelling and demyelination of the optic nerve. Examination of the cerebrospinal fluid may be helpful to check for markers of demyelinating disease, such as oligoclonal bands and anti-NMO antibodies. Results of ophthalmoscopic examination may be normal at the onset of symptoms if neuritis is primarily retrobulbar [7, 8].

Etiologic factors in cases of retrobulbar optic neuritis may be local, such as inflammation associated with sinusitis, or general, such as multiple sclerosis and undulant fever. Acute type is due to virus infection of the central nervous system. Some of the differential diagnoses of retrobulbar optic neuritis are including multiple sclerosis, neuromyelitis optica, idiopathic optic neuritis, and ischemic optic neuritis. Color vision loss is roughly equivalent in severity to visual acuity loss, whereas in optic neuritis, the disturbance of color vision is greater than that of visual acuity. Ophthalmoscopic examination reveals diffuse or partial swelling of the optic disk. This examination can also show papilledema or flame-shaped hemorrhage lesions in some pathogenesis such as traumatic optic neuropathy, toxic optic neuropathy, and psychogenic blindness [9, 10].

The severity of visual loss and degree of response to treatment can vary, but patients usually benefit from steroid treatment. Another recommended treatment for inflammatory ocular manifestation of BD is azathioprine. For resistant ocular manifestation, anti-TNF-*α* and alpha interferon are considered. TNF-*α* is known to be elevated in active BD [8, 11].

Our patient had been treated with intravenous immunoglobulin due to Kawasaki disease (KD) when she was three years; actually, this was the first sign of BD [12]. Also, in this patient, HLA B5 and HLA-B51 were positive. Endemic KD is associated with HLA-B51, while epidemic KD is not [13].

## 4. Conclusion

Although acute optic neuritis has been rarely reported in patients with Behçet disease (BD), our experience suggests that retrobulbar optic neuritis may be the first manifestation of neuro-BD.

## Abbreviations

BD: Behçet disease
GI: Gastrointestinal
HLA: Human leukocyte antigen
MOG: Myelin oligodendrocyte glycoprotein
MRI: Magnetic resonance imaging
NMO: Neuromyelitis optica
TNF: Tumor-necrotizing factor
KD: Kawasaki disease.
